# PTH-Related Protein Assays in Advanced Kidney Disease: Implications for Evaluation of Hypercalcemia

**DOI:** 10.1155/2023/6678658

**Published:** 2023-09-19

**Authors:** Jobira A. Woldemichael, Andres D. Pirela, Barry I. Freedman

**Affiliations:** Department of Internal Medicine, Section on Nephrology, Wake Forest University School of Medicine, Winston-Salem, NC, USA

## Abstract

Hypercalcemia is a common and potentially serious electrolyte abnormality that is often observed in patients with chronic kidney disease (CKD). When malignancy is considered, parathyroid hormone-related protein (PTHrP) levels are often measured. PTHrP is produced by cancer cells and mimics the effects of parathyroid hormone (PTH) to elevate serum calcium concentrations. The amino and carboxy termini of PTHrP are of functional relevance. C-terminal PTHrP levels accumulate with CKD and can be elevated in normocalcemic CKD patients who lack malignancy. The existence of amino (N)-terminal and carboxy (C)-terminal PTHrP assays and how their concentrations are impacted by CKD are reviewed herein. The case of a patient on maintenance hemodialysis who developed prolonged hypercalcemia with elevated PTHrP concentrations is presented. The workup revealed suppressed intact PTH, low 25-hydroxyvitamin D, and 1,25-dihydroxyvitamin D levels. The initial PTHrP assay returned elevated. However, it was unappreciated that it was the C-terminal assay and the patient underwent an unnecessary search for malignancy. A subsequent N-terminal PTHrP assay returned within the normal range. Many commercial labs run the C-terminal PTHrP assay as their first-line test. This can lead to inaccurate differential diagnoses in hypercalcemic patients with CKD. We emphasize the need to specifically request N-terminal PTHrP assays in patients with advanced kidney disease when humoral hypercalcemia of malignancy is suspected.

## 1. Introduction

Hypercalcemia is a common and potentially serious electrolyte abnormality that is often observed in patients with chronic kidney disease (CKD). Parathyroid hormone (PTH) is the primary regulator of serum calcium concentration via effects on the bone, kidney, and intestine [1]. Although the differential diagnosis of hypercalcemia can be broad, malignancy-associated hypercalcemia was recognized shortly after methods for measuring serum calcium became clinically available in the 1920s [2]. A novel radioimmunoassay for PTH in the 1970s suggested that the immunoreactivity in tumor cells and plasma from hypercalcemic patients with cancer differed from native forms of PTH [3]. Approximately 10 years later, purification of plasma culminated in discovery of parathyroid hormone-related protein (PTHrP) [2]. PTHrP is produced by cancer cells and mimics the effects of PTH. It is most commonly produced by squamous cell cancer of the head and neck, esophageal, cervical, lung, renal, ovarian, endometrial, and breast cancer, and HTLV-associated lymphoma [4].

PTHrP includes three distinct peptides: N-terminal, midregion, and C-terminal secretary forms. Each peptide is believed to have its own physiologic function. PTHrP, via a common PTH/PTHrP receptor (type 1 PTH receptor), mediates bone resorption and renal calcium reabsorption. The established and emerging functions of PTHrP in different organs along with the documented existence of effects in the midregion and carboxy termini suggest additional and yet unidentified receptors recognize these peptides [5].

PTHrP belongs to a family of endocrine factors that share a highly conserved N-terminal region (amino acids 1–34) and play key roles in calcium homeostasis, bone formation, and skeletal development [6]. Cloning of PTHrP complementary DNA (cDNA) revealed that 8 of the first 13 residues are identical to PTH, with the remaining identities no more than expected by chance [3]. As is the case for PTH, the structural requirements for full biological activity of PTHrP are contained within the first 34 amino acids.

Clinicians are aware that different PTH assays are commercially available and that the presence of CKD affects their concentrations. The existence of amino (N)-terminal and carboxy (C)-terminal PTHrP assays and how their concentrations are impacted by CKD are less widely appreciated. Herein, we present the case of a patient on maintenance hemodialysis, who developed prolonged hypercalcemia with an elevated PTHrP concentration. He underwent an unnecessary evaluation to exclude malignancy because it was not initially appreciated that elevated PTHrP was based on the C-terminal assay.

## 2. Case Presentation

A 30-year-old Hispanic male with type 2 diabetes mellitus and end-stage kidney disease (ESKD) treated with thrice weekly hemodialysis developed persistent asymptomatic hypercalcemia one month after initiating renal replacement therapy. Serum calcium concentrations ranged from 2.61 to 2.77 mmol/L (normal 2.18–2.58 mmol/L) on eight occasions over a 3-month period, with a normal serum albumin concentration. His bicarbonate-based dialysis bath contained 2.0 mmol/L potassium and 1.0 mmol/L calcium concentrations. During the period of hypercalcemia, serum phosphorous levels ranged from 1.45 to 1.94 mmol/L (normal 0.81–1.45 mmol/L) and intact PTH concentration was <0.64 pmol/L (normal 1.6–9.3 pmol/L). In addition, 25-hydroxyvitamin D and 1,25-dihydroxyvitamin D concentrations were 54.91 nmol/L (normal 74.9–249.6 nmol/L) and <12 pmol/L (normal 50.4–156.0 pmol/L), respectively. No other patient in the dialysis bay developed hypercalcemia, and the affected individual denied taking vitamins or multivitamins, excessive dietary dairy intake, prescription, or over-the-counter calcium or vitamin D supplements, and he lacked weight loss, anorexia, or signs suggestive of malignancy. He was not receiving 1,25-dihydroxyvitamin D, active vitamin D analogues, cinacalcet, or etelcalcetide with dialysis.

Table 1 summarizes laboratory values during the period of hypercalcemia. It reveals suppressed intact PTH, low 25-hydroxyvitamin D, and 1,25 (OH) dihydroxyvitamin D levels during the period that hypercalcemia was present. Thyroid-stimulating hormone (TSH) was normal at 2.68 mIU/L. Serum and urine electrophoresis was without monoclonal protein. A PTHrP assay was ordered and returned elevated at 110.4 pmol/L (normal 33.6–64.8 pmol/L); however, it was unappreciated that it was the C-terminal assay. Due to concern over malignancy in the setting of elevated PTHrP, a positron emission tomography (PET) scan was ordered and did not reveal a source of malignancy. A review of recent computed tomography scans and chest X-rays did not detect lesions suspicious for cancer.

An endocrinology consultation was requested for hypercalcemia in the setting of elevated PTHrP. The performance of an N-terminal PTHrP assay was recommended due to concern over the accumulation of C-terminal fragments of PTHrP in patients with advanced kidney disease. The N-terminal PTHrP assay returned within the normal range at 2 pmol/L (reference range less than 4.2 pmol/L), and hypercalcemia resolved spontaneously. Hypercalcemia has not recurred for 2 years, and the patient remains well.

## 3. Discussion

This case emphasizes the need to specifically request an N-terminal PTHrP assay in patients with advanced kidney disease when humoral hypercalcemia of malignancy (HHM) is being considered. Significantly higher levels of circulating C-terminal PTHrP are present in healthy individuals, compared to N-terminal PTHrP (ratio 1 : 14.4) [7]. Because of the robust signal and significantly higher levels of C-terminal (vs. N-terminal) PTHrP, its ease of detection, and the stability of C-terminal PTHrP fragments in blood, many clinical laboratories offer C-terminal detection as their primary screening assay for HHM. Unfortunately, this assay is more likely to be influenced by reduced kidney function [7].

Marked structural similarity exists in the initial (N-terminal) 34 amino acids of PTH and PTHrP, where the major biologic activity resides. This is likely the result of an evolutionary relationship between these molecules that are likely derived from a common ancestral gene and evolved due to gene duplication [6]. Characteristic proteolytic sites within the human PTHrP protein are also conserved, and these generate three distinct peptides: N-terminal PTHrP (1–36), midregion PTHrP (38–94), and C-terminal PTHrP (109–138) [6]. PTHrP and PTH share marked sequence and conformational homologies between their 1–34 NH_2_ termini, which explains their ability to bind with equal affinity to a single common PTH/PTHrP receptor, PTH receptor type 1.

Kidney disease impacts the metabolism of both PTH and PTHrP [8]. Patients with HHM have increased N-terminal and C-terminal PTHrP levels. However, C-terminal immunoreactive PTHrP levels may also be elevated in normocalcemic patients with CKD who lack malignancy. In these cases, amino (N)-terminal iPTHrP levels are normal. Among 25 patients with CKD and creatinine clearances <5 to 66 ml/min/1.73 m^2^ from diverse etiologies, all had low or undetectable intact (i) PTHrP concentrations (1–74) [7]. In contrast, iPTHrP (109–138) concentrations were undetectable in patients with a creatinine clearance ≥20 ml/min/1.73 m^2^ and rose in patients with creatinine clearances <20 ml/min/1.73 m^2^ [9]. The mean levels of iPTHrP (109–138) were slightly higher in patients on hemodialysis than in patients on chronic ambulatory peritoneal dialysis, suggesting greater peritoneal clearance of the C-terminal peptide [9]. Studies in patients with HHM demonstrated elevations of separate amino- and carboxy-terminal immunoreactive PTHrP fragments in patients using both a two-site immunoradiometric assay (IRMA) with amino-terminal specificity (PTHrP (1–74)) also known as (N-terminal PTH-RP) and with a carboxy-terminal-specific radioimmunoassay (RIA) for PTHrP (109–138) [9]. The excretion of carboxy-terminal iPTHrP peptides in the urine of hypercalcemic and normocalcemic patients with cancer has been reported [10].

We present a patient with ESKD on hemodialysis with hypercalcemia and an elevated C-terminal PTHrP concentration that led to an unnecessary search for malignancy. Approximately 20–30% of patients with malignancy develop hypercalcemia, and 80% have HHM with elevated levels of PTHrP. PTHrP concentrations should be measured in such patients when the diagnosis cannot be made on clinical grounds or when the cause of hypercalcemia is uncertain. It is critically important that nephrologists be aware that only the N-terminal PTHrP assay be ordered in patients with stages 4-5.

## Figures and Tables

**Table 1:** Serum chemistries during the 3-month phase of hypercalcemia.

	12/6/20	1/7/21	2/4/21	2/7/21	2/27/21	3/4/21	3/9/21	3/11/21	3/16/21	4/8/21
Ca (mmol/L) normal 2.18–2.58	1.85	2.62	2.74		2.62	2.74	2.77	2.77	2.67	2.67
Phos (mmol/L) normal 0.81–1.45	1.26	1.65	2			1.71		1.65	1.81	1.48
25(OH)D normal 74.9–249.6								54.91		
1,25(OH)_2_ (pmol/L) normal 50.4–156.0								<12		
iPTH (pmol/L), C-terminal normal 1.6–9.3	1.59	<0.64	<0.64							<0.64
Albumin (mmol/L) normal 0.87–1.25	0.42								0.65	
Light chain ratio									1.96	
Ionized calcium (mmol/L) normal 1.05–1.3	0.29									
PTHrP (pmol/L) normal 33.6–64.8									110.4	
